# Reichel Syndrome in Children: A Case Report

**DOI:** 10.1055/s-0044-1779511

**Published:** 2024-12-27

**Authors:** Hanene Lassoued Ferjani, Hiba Bettaib, Ben Nessib Dorra, Kaouther Maatallah, Mourad Jenzri, Wafa Hamdi

**Affiliations:** 1 Departamento de Reumatologia, Kassab Orthopedics Institute, Ksar Saïd, Tunísia; 2 Unidade de pesquisa UR17SP04, 2010, Ksar Said 2010, Tunis, Tunísia; 3 Departamento de Ortopedia Pediátrica, Kassab Orthopedics Institute, Ksar Saïd, Tunísia

**Keywords:** chondromatosis, pediatrics, shoulder, synovial

## Abstract

Reichel syndrome or primary synovial chondromatosis (PSC) is an uncommon benign metaplastic condition that usually affects large joints. Though shoulder involvement was scarce, there are only a few cases in the pediatric population. A 14- year-old boy was admitted to the Pediatric Orthopedics department with right shoulder pain for 14 months. Imaging revealed multiple loose bodies distributed throughout the glenohumeral joint. Upon the arthroscopic approach, we remove all cartilaginous nodules within the glenohumeral space and abarticular tendon. Histopathologic examination confirmed the diagnosis of primary synovial chondromatosis. At follow-up, the patient remains free of symptoms, and shoulder radiographs showed no recurrence of calcification. The present case illustrates the clinical patterns, imaging features, histological findings, and therapeutic management of shoulder primary synovial chondromatosis in a pediatric patient.

## Introduction

Reichel syndrome or primary synovial chondromatosis (PSC) is a benign tumor with cartilaginous nodules in the synovium joints. 1 2 3 The glenohumeral joint is an unusual location in PSC, particularly in pediatric patients. 3 4 5 To our knowledge, there are only 5 cases of children reported in the literature. Herein, we report a rare case of PSC in a 14-year-old boy with an uncommon localization in the shoulder revealed by the chronic pain and the limited motion in the right arm.

## Case Report

A 14-year-old, right-handed boy presented to the Pediatric Orthopedics department with right shoulder pain. He complained for 14 months of a history of pain and discomfort in his right shoulder.

He reported no symptoms of weight loss, fatigue, systemic signs, or any other arthralgia.

On physical examination, there was no obvious deformity or atrophy involving the affected shoulder. We noted a decreased range of motion, in comparison to the uninvolved side, with respectively: flexion to 160°, extension to 40°, abduction to 140°, adduction to 40°, internal rotation to L4, and external rotation to 50°. Subacromial impingement signs, as well as rotator cuff tear tests, were negative.

**Fig. 1:**
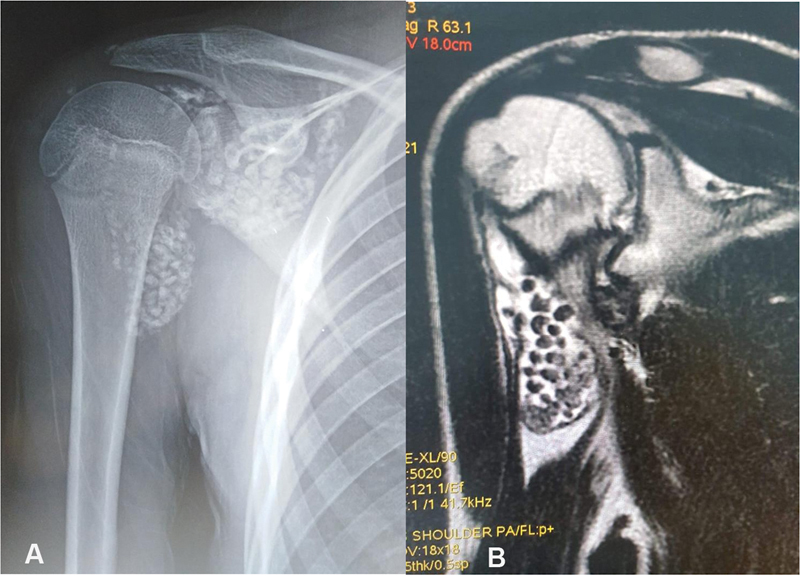
**A** ) radiograph of the right shoulder demonstrates the presence of numerous radiopaque loose bodies in the glenohumeral joint. **B** ) STIR magnetic resonance image shows an excess of synovial joint fluid and multiple centers of nodular calcification around the joint and within the biceps tendon shea.

Plain radiographs showed multiple radio-opaque bodies distributed throughout the glenohumeral joint, without bone defection or joint narrowing ( Fig. 1A ). Subsequent Magnetic resonance imaging (MRI) revealed a high number of calcified intra-articular loose bodies around the joint and the biceps tendon ( Fig. 1B ). PSC was strongly suspected.

**Fig. 2:**
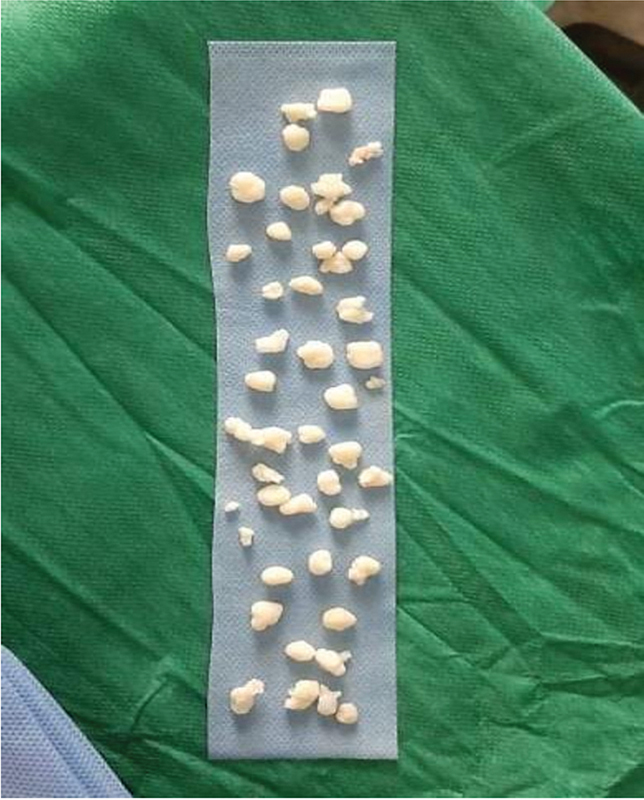
Numerous synovial Chondromatosis nodules removed from the shoulder joint (size between 10 and 15 mm).

**Fig. 3:**
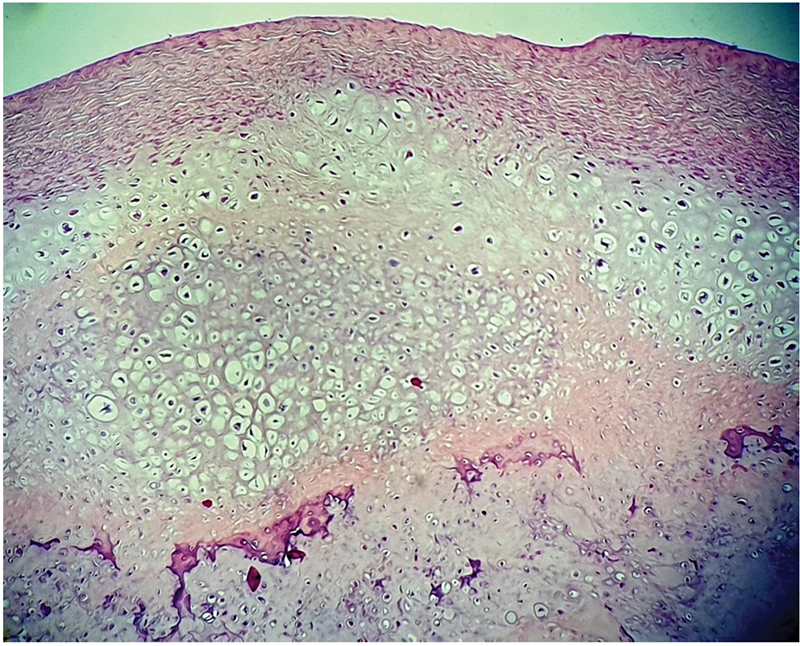
Histological image of the lesion showing multiple cartilaginous nodules composed of clustered chondrocytes, embedded in synovium. Note the presence of focal calcification and enchondral ossification (small arrow). The nodules are surrounded by a rim of residual synovial tissue (large arrow). (hematoxylin-eosin, original magnification × 400).

We choose a shoulder arthroscopy using a deltopectoral approach to remove the tumors nodules. More than 50 shiny and solid bodies, with an average size of 10–15 mm, were retrieved ( Fig. 2 ). There were also several bodies within the coracoid process and the conjoint tendon. The synovial tissue, bursas, and cartilages appeared intact. Upon removing the particles, we complete a partial synovectomy to avoid the relapse. Histology of loose bodies and synovium confirmed the diagnosis of PSC without any evidence of malignant transformation ( Fig. 3 ).

Postoperative shoulder X-rays did not show any densities. The patient was discharged after the surgery using the arm sling. At three months of follow-up, the patient remains free of symptoms, and shoulder radiographs showed no recurrence of calcification.

## Discussion

First mentioned by Jaffe et al., 1 PSC is a rare benign tumor affecting the synovial cavity. It is a proliferation of multiple cartilaginous nodules in the synovium of joints, tendon sheaths, and bursae.

According to the literature, this disorder usually occurs in men between the ages of 30 and 50 old-years. It has been reported that the knees, hip, elbow, and wrists are the main affected joints in descending order of frequency. 6 7 The involvement of the shoulder is unusual in adults, and more exceptional during childhood. To the best of our knowledge, only five cases have been reported in the literature 3 4 5 7 ( Table 1 ).

**Table 1:** Clinical presentation, treatment, and outcome in patients with Synovial Chondromatosis in literature

	Age	Sex	Duration of symptom (months)	Trigger factor	Clinical presentation	Surgical option	Follow-up Period (months)	Recurrence
**Nashi et al.1998** . 10	14	Male	6	Sporting activities	- Shoulder pain	Under observation	24	−
**Miranda et al.** **2004** . 7	10	Female	1	Sporting activities	- Shoulder pain - Discomfort	Arthrotomy and synovectomy	12	No
**Hamada et al.** **2005** . 4	14	Female	18	Sporting activities	- Shoulder pain - Discomfort	Arthroscopy	36	No
**Kirchoff et al.** **2008** . 3	14	Male	12	No	- Shoulder pain - Palpable mass	Arthrotomy and synovectomy	9	No
**Sinikumpu et al.** **2020** . 5	14	Male	12	Sporting activities	- Shoulder pain - Stiffness - Palpable mass	Arthrotomy and synovectomy	12	No
**The present case** **2021**	14	Male	14	No	- Shoulder pain - Discomfort	Arthroscopy and synovectomy	5	No

Based on the underlying pathogenesis, synovial chondromatosis may be primary or secondary. The PSC, called idiopathic synovial osteochondromatosis or Reichel syndrome, usually occurs in a previously healthy joint.

In contrast, secondary osteochondromatosis is a sequela of intra-articular pathology as osteochondral fracture, osteochondritis dissecans, and osteoarthritis. 4 6 8

In our case, the young-onset, the absence of a history of trauma, and the unremarkable results of blood tests strengthen the diagnosis of PSC.

It is noteworthy that histopathologic analysis is mandatory to distinguish between these conditions. 4 6 9 In a series of 136 presumed synovial osteochondromatosis, Villain et al showed that the histopathological patterns are different. In the present case, the histologic evaluation revealed multiple cartilaginous nodules arranged in clusters and embedded in the synovium. 9

Clinical presentation is often nonspecific. 2 3 6 As a result, patients may experience long symptoms delays before the final diagnosis. Like the current case, most children with PSC of the shoulder were diagnosed between 10 to 14 years old with a diagnosis delay ranging from 1 to 18 months from symptoms onset. 3 4 5 7

In a recent literature review of cases with osteochondromatosis occurring in the shoulder, the most common reported symptoms were mainly shoulder pain, uncomfortable feeling during exercises, and locked joint movement. 7 Our report is the following data in the literature. Interestingly, a palpable bony mass may occur, as described in two children with PSC of the shoulder. 3 5

Radiographic features vary according to the degree of ossification. In the later stages of the disease, the plain radiographs showed a characteristic image with multiple intraarticular radio-opacities. These calcifications are frequently very similar and uniform in size with a typical nest-like arrangement. Thus, plain radiographs may be normal in the earlier stages (30% of cases). 8 Sometimes, the diagnosis overlaps between differential diagnostics such as osteosarcoma and chondrosarcoma.

Hence, MRI plays a pivotal role in confirming the diagnosis by revealing intrasynovial hypointense nodules on T1 and T2-weighted images. MRI also aids in the management of surgical approaches. 2 3 5

The optimal therapeutic management of the disease requires surgical removal of any loose bodies. 3 6 7 8 Partial synovectomy, optional but often recommended, may decrease the recurrence rate. 5 6 8 Histopathological analysis of the loose bodies and synovial tissue is mandatory as a malign transformation may occur in up to 5%. 2 3

The choice of surgical procedure is still a matter of debate. 6 Open surgery remains the mainstay of treatment and is highly recommended in cases of osteochondromatosis with soft-tissue involvement and limited anatomic space access. 3 Moreover, this approach was often preferable in pediatric patients with shoulder involvement. 3 5 10 In line with Hamada et al, we opted for shoulder arthroscopy using the deltopectoral approach. 4

According to the literature, recurrence is common and ranges between 15% and 30%. 5 6 It's noteworthy to mention that no recurrence of calcification was reported among pediatric patients with PSC of the shoulder. 3 5 10 In our case, the short duration of follow-up was not sufficient to make a definitive conclusion.

The present case illustrates a rare entity of PSC that combined the intra and extraarticular involvement of the shoulder. MRI is a powerful key for early diagnosis. The management of this affection, like in adult patients, is based on the chondromyxoid bodies removed through open or arthroscope-assisted surgery. Histological analysis is mandatory since a malign transformation might occur.
