# Supplementary material for: Reichel Syndrome in Children: A Case Report
Source: Rev Bras Ortop (Sao Paulo). 2024 Dec 27;59(Suppl 2):e212–5. doi: 10.1055/s-0044-1779511 (PMC11679629; doi:10.1055/s-0044-1779511)
Supplement: Related texts

# Research article: Síndrome de Reichel em crianças: Relato de caso

**Keywords:** condromatose, ombro, pediatria, sinovial

## Abstract

A síndrome de Reichel ou condromatose sinovial primária (CSP) é uma metaplasia benigna incomum geralmente observada em grandes articulações. O acometimento do ombro é raro e há apenas alguns casos na população pediátrica. Um menino de 14 anos foi atendido no departamento de Ortopedia Pediátrica com dor no ombro direito há 14 meses. Os exames de imagem revelaram múltiplos corpos livres distribuídos por toda a articulação glenoumeral. À abordagem artroscópica, removemos todos os nódulos cartilaginosos no interior do espaço glenoumeral e tendão abarticular. O exame histopatológico confirmou o diagnóstico de CSP. Ao acompanhamento, o paciente não apresenta sintomas e as radiografias do ombro não mostraram recidiva da calcificação. Este caso ilustra os padrões clínicos, as características em técnicas de diagnóstico por imagem, os achados histológicos e o manejo terapêutico da CSP no ombro de um paciente pediátrico.

## Introdução

A síndrome de Reichel ou condromatose sinovial primária (CSP) é um tumor benigno com nódulos cartilaginosos nas articulações sinoviais. 1 2 3 A articulação glenoumeral é uma localização incomum da CSP, principalmente em pacientes pediátricos. 3 4 5 Até onde sabemos, existem apenas cinco casos de crianças relatados na literatura. Aqui, relatamos um caso raro de CSP em um menino de 14 anos com localização incomum no ombro, revelado por dor crônica e limitação do movimento do braço direito.

## Relato de Caso

Um menino de 14 anos, destro, foi atendido no departamento de Ortopedia Pediátrica com dor no ombro direito. O paciente queixava-se de dor e desconforto no ombro direito há 14 meses.

Não relatou sintomas de perda de peso, fadiga, sinais sistêmicos ou qualquer outra artralgia.

Ao exame físico, não havia deformidade ou atrofia óbvia no ombro acometido. Notamos uma diminuição da amplitude de movimento em comparação ao lado não acometido, respectivamente de 160° de flexão, 40° de extensão, 140° de abdução, 40° de adução, rotação interna em L4 e 50° de rotação externa. Os sinais de impacto subacromial, bem como os testes de ruptura do manguito rotador, foram negativos.

Radiografias simples mostraram múltiplos corpos radiopacos distribuídos por toda a articulação glenoumeral, sem defeito ósseo ou estenose articular ( Fig. 1A ). A ressonância magnética (RM) subsequente revelou um número elevado de corpos livres intra-articulares calcificados ao redor da articulação e do tendão do bíceps ( Fig. 1B ). Havia forte suspeita de CSP.

**Fig. 1:** **A** ) A radiografia do ombro direito demonstra a presença de numerosos corpos livres radiopacos na articulação glenoumeral. **B** ) A imagem de ressonância magnética STIR mostra um excesso de fluido sinovial articular e múltiplos centros de calcificação nodular ao redor da articulação e no interior da bainha do tendão do bíceps.

Optamos por uma artroscopia do ombro por abordagem deltopeitoral para remoção dos nódulos tumorais. Mais de 50 corpos brilhantes e sólidos, com tamanho médio de 10 a 15 mm, foram retirados ( Fig. 2 ). Também havia diversos corpos no interior do processo coracoide e do tendão articular. O tecido sinovial, as bursas e cartilagens pareciam intactos. Ao remover as partículas, realizamos uma sinovectomia parcial para evitar recidivas. A histologia dos corpos livres e da sinóvia confirmou o diagnóstico de CSP sem qualquer evidência de transformação maligna ( Fig. 3 ).

**Fig. 2:** Numerosos nódulos sinoviais de condromatose removidos da articulação do ombro (tamanhos entre 10 e 15 mm).

**Fig. 3:** A imagem histológica da lesão revela múltiplos nódulos cartilaginosos compostos por condrócitos agrupados, embutidos na sinóvia. Observe a presença de calcificação focal e ossificação endocondral (seta pequena). Os nódulos são circundados por uma borda de tecido sinovial residual (seta grande). (Hematoxilina-eosina, aumento original × 400).

As radiografias pós-operatórias do ombro não mostraram quaisquer densidades. O paciente recebeu alta após a cirurgia usando uma tipoia no braço. Aos 3 meses de acompanhamento, o paciente continua sem sintomas e as radiografias do ombro não mostraram recidiva da calcificação.

## Discussão

Descrita pela primeira vez por Jaffe et al., 1 a CSP é um tumor benigno raro que afeta a cavidade sinovial. É uma proliferação de múltiplos nódulos cartilaginosos na sinóvia das articulações, bainhas dos tendões e bursas.

De acordo com a literatura, esse distúrbio é geralmente observado em homens entre 30 e 50 anos de idade. As principais articulações acometidas, em ordem decrescente de frequência, são joelhos, quadril, cotovelo e punhos. 6 7 A CSP de ombro é incomum em adultos e ainda mais excepcional durante a infância. Até onde sabemos, apenas cinco casos foram relatados na literatura 3 4 5 7 ( Tabela 1 ).

**Tabela 1:** Quadro clínico, tratamento e desfecho em pacientes com condromatose sinovial na literatura

	Idade	Sexo	Duração dos sintomas (meses)	Fator desencadeante	Quadro clínico	Opção cirúrgica	Período de acompanhamento (meses)	Recidiva
**Nashi et al.1998** . 10	14	Masculino	6	Atividades esportivas	- Dor no ombro	Sob observação	24	−
**Miranda et al.** **2004** . 7	10	Feminino	1	Atividades esportivas	- Dor no ombro - Desconforto	Artrotomia e sinovectomia	12	Não
**Hamada et al.** **2005** . 4	14	Feminino	18	Atividades esportivas	- Dor no ombro - Desconforto	Artroscopia	36	Não
**Kirchoff et al.** **2008** . 3	14	Masculino	12	Ausente	- Dor no ombro - Massa palpável	Artrotomia e sinovectomia	9	Não
**Sinikumpu et al.** **2020** . 5	14	Masculino	12	Atividades esportivas	- Dor no ombro - Rigidez - Massa palpável	Artrotomia e sinovectomia	12	Não
**Este caso** **2021**	14	Masculino	14	Ausente	- Dor no ombro - Desconforto	Artroscopia e sinovectomia	5	Não

Com base em sua patogênese, a condromatose sinovial pode ser primária ou secundária. A CSP, denominada osteocondromatose sinovial idiopática ou síndrome de Reichel, é geralmente observada em uma articulação outrora saudável.

Por outro lado, a osteocondromatose secundária é uma sequela de uma patologia intra-articular, como fratura osteocondral, osteocondrite dissecante e osteoartrite. 4 6 8

No nosso caso, o início na adolescência, a ausência de um histórico de traumatismo e os achados normais em exames de sangue reforçam o diagnóstico de CSP.

Vale ressaltar que a análise histopatológica é obrigatória para distinguir essas doenças. 4 6 9 Em uma série de 136 casos de suposta osteocondromatose sinovial, Villain et al. mostraram diferentes padrões histopatológicos. No presente caso, a avaliação histológica revelou múltiplos nódulos cartilaginosos dispostos em aglomerados e embutidos na sinóvia. 9

De modo geral, o quadro clínico é inespecífico. 2 3 6 Assim, o diagnóstico final pode demorar muito após o início dos sintomas. Como no caso aqui descrito, a maioria das crianças com CSP do ombro foi diagnosticada entre 10 e 14 anos de idade, com diagnóstico 1 a 18 meses depois do início dos sintomas. 3 4 5 7

Em recente revisão de literatura de casos com osteocondromatose no ombro, os sintomas mais relatados foram dor no ombro, desconforto durante exercícios e redução do movimento articular. 7 Nosso relato é o seguinte na literatura. Curiosamente, uma massa óssea palpável pode ser observada, como ocorreu em duas crianças com CSP do ombro. 3 5

As características radiográficas variam de acordo com o grau de ossificação. Nos estágios mais avançados da doença, as radiografias simples mostraram uma imagem característica com múltiplas radiopacidades intra-articulares. Essas calcificações tendem a ser muito semelhantes e de tamanho uniforme, em disposição típica de ninho. Assim, as radiografias simples podem ser normais nos primeiros estágios (30% dos casos). 8 Às vezes, há sobreposição de diagnósticos diferenciais, como osteossarcoma e condrossarcoma.

Logo, a RM desempenha um papel fundamental na confirmação do diagnóstico, revelando nódulos hipointensos intrassinoviais nas imagens ponderadas em T1 e T2. A RM também auxilia o manejo das abordagens cirúrgicas. 2 3 5

O manejo terapêutico ideal da doença requer a remoção cirúrgica de quaisquer corpos livres. 3 6 7 8 A sinovectomia parcial, opcional, mas bastante recomendada, pode diminuir a taxa de recidiva. 5 6 8 A análise histopatológica dos corpos livres e do tecido sinovial é obrigatória, já que a frequência de transformação maligna chega a 5%. 2 3

A escolha do procedimento cirúrgico ainda é motivo de debate. 6 A cirurgia aberta continua sendo a base do tratamento e é altamente recomendada em casos de osteocondromatose com acometimento de partes moles e acesso limitado ao espaço anatômico. 3 Além disso, essa abordagem foi frequentemente preferível em pacientes pediátricos com doença no ombro. 3 5 10 Em consonância com Hamada et al., optamos pela artroscopia do ombro pela abordagem deltopeitoral. 4

De acordo com a literatura, a recidiva é comum, com frequência entre 15% e 30%. 5 6 Vale ressaltar que ainda não há relatos de recidiva da calcificação entre os pacientes pediátricos com CSP do ombro. 3 5 10 Em nosso caso, a curta duração de acompanhamento não foi suficiente para uma conclusão definitiva.

Este caso ilustra uma doença rara, a CSP, com acometimento intra e extra-articular do ombro. A RM é uma ferramenta poderosa para o diagnóstico precoce. O manejo dessa afecção, como em pacientes adultos, baseia-se na remoção dos corpos condromixoides por meio de cirurgia aberta ou artroscópica. A análise histológica é obrigatória devido à possível transformação maligna.
